# Clonal Diversity in Multi Drug Resistant (MDR) Enterococci Isolated from Fecal Normal Flora

**Published:** 2015

**Authors:** Meysam Hasannejad Bibalan, Morteza Eshaghi, Javad Sadeghi, Mahla Asadian, Tahmineh Narimani, Malihe Talebi

**Affiliations:** 1 *Department of Microbiology, Faculty of Medicine, Iran University of Medical Sciences, Tehran, IR Iran.*; 2 *Department of Microbiology, School of Medicine, Tehran University of Medical Sciences, Tehran, IR Iran.*

**Keywords:** Enterococcus, Rep- PCR, antibiotic profile, normal flora

## Abstract

Enterococci are Gram positive and catalase- negative cocci that are found in the gastrointestinal tract of mammals and birds, and are readily isolated from soil, surface and waters. The aim of this study was to discriminate between *Enterococcus* isolates based on repetitive element sequence based –PCR (Rep-PCR) with the BOXA2R primer and their antibiotics profile. *Enterococci* isolates were obtained from 180 fecal samples. The isolates were identiﬁed by biochemical reaction and specific identification was confirmed by PCR with species specific primers. All isolates were subjected to Rep typing and antimicrobial susceptibility tests. Rep-PCR analysis of 180 isolates revealed 93 REP types with forty-five single types (ST1 to ST45) and forty-eight common types (CT1 to 48). Antibiotic susceptibility tests exhibited that 53 (29.4%), 43 (23.8%), 11 (6.1%) and 9 (5%) were resistant to erythromycin, tetracycline, gentamicin and ciprofloxacin respectively but among the isolates, sixteen were multi drug resistant (MDR). These MDR isolates showed 11 Rep types with seven single types and four common types. In addition, 81.2% of MDR isolates were from male subjects and the average age of these persons was more than fifty years. This study showed that 56.2% of MDR isolates were homogeneous with 95 % similarity, and high rate of resistance to tetracycline and erythromycin (81.2%) were observed in these isolates. The concern about these normal flora isolates are the pathogenic potential of these bacteria through the horizontal transfer of antibiotic resistance and virulence genes.

Enterococci are Gram positive, aerotolerant fermentative and catalase-negative cocci that are found in the gastrointestinal (GI) tract of mammals, birds and are readily isolated from soil, surface waters and sediments. The ability of this organism to survive in various host environments provides great temptation to be selected as a fecal contaminant indicator (1).

On the other hand, they are becoming signiﬁcant pathogens worldwide, especially with respect to nosocomial infections (2). Multiple antibiotic exposure in hospital settings, promotes a high level of resistance to glycopeptides and aminoglycosides, which are of great clinical importance, providing them with an evolutionary pressure for selective advantage (3).

Among *enterococcus* species, *E. faecalis* and *E. Faecium* are important and have been isolated from healthy persons and patients. These species are causative agents of a variety of human infections such as bloodstream, heart, abdomen and urinary tract infections (4).

Differentiation of enterococci at the species level is mostly made by phenotypic methods, such as detecting the utilization of some carbohydrates and other substrates (5). In recent years, molecular techniques such as ribotyping, pulsed-field gel electrophoresis, Phene Plate (PhP) typing and repetitive element sequence based –PCR (Rep-PCR) have been used as effective methods for detecting and discriminating of pathogenic as well as non pathogenic isolates (1).

Among these methods, Rep-PCR is a DNA ﬁngerprint technique that uses repetitive intergenic DNA sequences to differentiate between sources of fecal pollution. In this technique, the DNA present between adjacent repetitive extragenic elements is ampliﬁed by PCR and various DNA fragment sizes are produced. The PCR products are separated based on their size by agarose-gel electrophoresis to yield ﬁngerprint patterns. In the final step, these patterns can be analyzed with the pattern recognition computer software (6,7).

Rep- PCR method is more accurate, reproducible and efﬁcient than some other typing methods such as ribotyping, PFGE and PhP typing (6). On the other hand, this method is suitable for microbial ecology and subtype analysis investigations (8).

In this study, Rep-PCR with the BOXA2R primers was used to discriminate between *enterococcus* isolates and their antibiotics profile was assessed.

## Materials and methods

**Sample collection and species identiﬁcation**

Enterococci isolates were obtained from 180 fecal samples (persons between 20 to 70 years old) during 2014 in Tehran, Iran. The isolates were identiﬁed by their growth on m-*enterococcus* agar (ME agar), Gram staining, catalase reaction and streaking on bile aesculin azid agar plates (9). Colonies with a black zone of aesculin hydrolysis were considered as presumptive enterococci. Final identification was confirmed by PCR with species specific primers (10).

**Antimicrobial susceptibility tests**

Resistance phenotypes were determined by disk diffusion method on Mueller Hinton agar according to the guidelines of the clinical and laboratory standards institute (CLSI) (11). Antibio-tic disc test, included vancomycin, ciprofloxacin, tetracycline, erythromycin, chloramphenicol, ampi-cillin, linezolid, teicoplanin, gentamycin and syner-cid (quinupristin and dalfopristin). *E. faecalis* ATCC 29212 was used as reference strain for antimicrobial susceptibility testing.

**DNA extraction and** **REP-PCR**

Bacterial DNA was prepared with DNA extraction kit (Qiagen, Hilden, Germany). BOX-PCR analysis was ideally performed using a BOXA2R oligonucleotide sequence (5′-ACG TGGTTTGAAGAGATTTTCG-3′) as described by Pangallo et al.(1). Briefly, PCR assay was performed in a total volume of 25 μl containing 50 ng DNA, 1.25 U Taq DNA polymerase, 1xPCR buffer, 2.5 mM MgCl_2_ ,200 μM dNTP, 30 pmol BOXA2 primer and using the following conditions: initial denaturation at 95 °C for 7 min, 35 cycles of denaturation at 90 °C for 30 s ,annealing at 40 °C for 1 min and elongation at 60 °C for 6 min with ﬁnal extension at 65 °C for 16 min. Electrophoresis of the PCR products was performed in 1% agarose gel for 150 min at 80 V followed by visualization in UV transilluminator after staining with ethidium bromide.

## Results

**Prevalence of enterococci species**

A total of 180 enterococci isolates were obtained from fecal samples. 96 (53.3%) and 84 (46.7%) of these isolates were from male and female subjects, respectively. Species identification showed that 158 (87.7%) and 22 (12.3%) of isolates were *E. faecium and E. faecalis*, respectively.

**Antibiotic resistance**

All of the 180 isolates (100%) were susceptible to chloramphenicol, ampicillin, linezolid, teicoplanin, vancomycin and synercid (quinupristin and dalfopristin) but antibiotic susceptibility tests exhibited that 53 (29.4%), 43 (23.8%), 11 (6.1%) and 9 (5%) of isolates were resistant to erythromycin, tetracycline, gentamicin and ciprofloxacin, respectively. Among these isolates, sixteen were multi drug resistant (MDR) with resistance to erythromycin and tetracycline (13 isolates), erythromycin and ciprofloxacin (2 isolates) and ciprofloxacin and tetracycline (1 isolate). Of the total MDR isolates, 14 (81.5%) were *E. faecium* and 2 (12.5%) were *E. faecalis*. 

Thirteen (81.2%) and three (18.8%) of MDR isolates were obtained from male and female subjects, respectively, and 62.5% of isolates were from persons aged more than fifty years.

**REP-PCR** **typing of** **MDR isolates**

REP-PCR analysis of 180 isolates showed 93 REP types with forty-five (25%) single types (ST1 to ST45) and forty-eight common types (CT1 to 48). Forty-eight common types included 135 (%75) isolates. Each common type was observed in 2 to 4 isolates. The diversity analysis of Simpson’s index showed REP-PCR with Di =0.96.

**Fig. 1:**
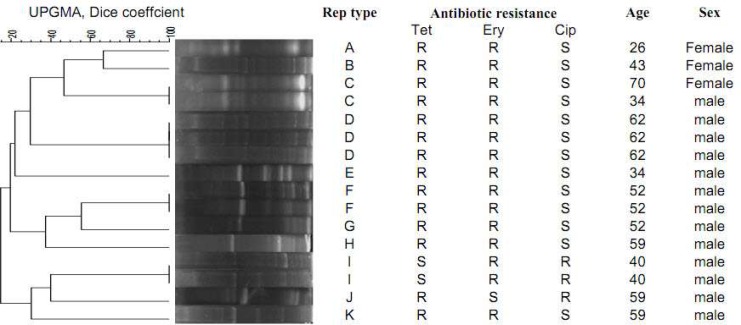
Dendrogram cluster analysis of REP-PCR data for 16 MDR Enterococcus isolates. Age, sex, Rep type and patterns are indicated. Cip: ciproﬂoxacin; Ery: erythromycin; Tet: tetracycline

On the other hand, the analysis of sixteen MDR isolates showed 11 Rep types with four common types covering 9 (56.2%) isolates with 95 % similarity. The remaining 7 isolates (43.8%) were highly divergent belonging to seven single types (ST). All REP types, except for CT I and ST J, were resistant to tetracycline and erytromycine. On the other hand, CT I was resistant to ciprofloxacin and erytromycine. In addition, ST J was resistant to ciprofloxacin and tetracycline (Figure 1).

## Discussion

The enterococci are members of the intestinal microﬂora and include more than 20 different species, but *E. faecalis *and *E. faecium *are dominant in clinical isolates, environment and foods (12).

In the present study, all of the isolates (100%) were susceptible to chloramphenicol, ampicillin, linezolid, teicoplanin, synercid (quinupristin and dalfopristin) and vancomycin. Susceptibility to vancomycin was also observed in other studies (13, 14). On the other hand, inconsistent with the results of Ali et al. in 2013 in Pakistan (15) and Kim et al. in 2013 in Korea (13), our results showed the resistance to erythromycin, tetracycline and ciprofloxacin. This may result from the choice of antibiotics and consumption dose that had an effect on antibiotic resistance worldwide.

Moreover, colonization and infection with MDR enterecocci- those strains with significant resistance to two or more antibiotics- occur worldwide. Acquiring plasmid- encoded antibiotic resistance genes and virulence factors are the concern for this MDR normal flora, because they can become pathogenic and may be very important in public health (16).

On the other hand, sixteen MDR isolates were obtained from all of the samples. High rate of resistance to tetracycline and erythromycin (81.2%) was observed in our MDR isolates, which is similar to a report from Pakistan (15). In addition, 81.2% of MDR isolates were from male subjects and the average age of these persons was more than fifty years.

Molecular techniques used for typing of bacteria are variable in terms of standardization, cost, reproducibility, discriminatory power and interpretation. REP-PCR, is a simple procedure to distinguish closely related organisms. This method is less expensive and faster than many other methods such as PFGE and AFLP typing (17).

In the current study, the analyzes showed 93 REP types. 45 (25%) of these isolates were single types (ST) and the other 135 (75%) isolates belonged to Forty-eight common types with the biggest cluster containing four isolates. Furthermore, Rep PCR with MDR isolates detected 11 Rep types with four common types covering 9 (56.2%) isolates with 95 % similarity. Also, some strains with different Rep types were isolated from the same person, showing the heterogeneity of normal flora enterococci.

Although BOX-PCR genotyping have discriminatory power, is cheap and reproducible, but the incorrect grouping of some strains and difficulties of pattern analysis are some of the limitations of this method. The concern about these normal flora isolates are the pathogenic potential of these bacteria through the horizontal transfer of antibiotic resistance and virulence genes.
